# Influence of parental employment status on Dutch and Slovak adolescents' health

**DOI:** 10.1186/1471-2458-6-250

**Published:** 2006-10-12

**Authors:** Maria Sleskova, Jolanda Tuinstra, Andrea Madarasova Geckova, Jitse P van Dijk, Ferdinand Salonna, Johan W Groothoff, Sijmen A Reijneveld

**Affiliations:** 1 Institute of Philological and Social Sciences, University of PJ Safarik, Kosice, Slovakia; 2 Research Institute of Culture, Welfare and Care, Province of Drenthe, The Netherlands; 3 Department of Social Medicine, University Medical Center Groningen, University of Groningen, The Netherlands

## Abstract

**Background:**

Recent research shows the possibility that the link between parental employment status and children's health can be affected by different cultural or societal settings. The aim of this study was to explore whether the effect of father's and mother's employment status on several aspects of adolescents' health differs between Slovakia and the Netherlands.

**Methods:**

Two data sets were used: 2616 Slovak adolescents (mean age 14.9) and 2054 Dutch adolescents (mean age 16.3). Self-rated health, GHQ-12, long-term well-being and Rosenberg self-esteem scale were used to assess the health of adolescents. Parental employment status was classified into the following categories: employed, unemployed, disabled, housewife (among mothers only). Logistic regression analyses were done separately for males and females.

**Results:**

Results indicate that having an unemployed father negatively influences self-rated health and long-term well-being of Slovak male adolescents, but has no effect on the health of Dutch adolescents. Secondly, having a disabled father has a negative effect on the psychological well-being of Dutch males and the self-rated health of females, but does not influence the health of Slovak adolescents. Thirdly, having a mother who is disabled, unemployed or a housewife has a negative effect on the self-esteem of Slovak adolescents. Fourthly, Dutch males whose mother was a housewife had worse long-term well-being than those with an unemployed mother, whereas Dutch females whose mother was a housewife reported better psychological well-being than those with an employed mother.

**Conclusion:**

To conclude briefly our results, father's unemployment seems to be a better predictor of health for Slovak adolescents, father's disablement of health for Dutch ones. Mother's employment status seemed to be important for the self-esteem of Slovak adolescents and mother as a housewife for the health of Dutch ones. This suggests that the link between parental employment status and the health of their children may vary between countries, and therefore further studies involving various cultures are needed.

## Background

Consequences of parental employment status on their children's health have usually been studied together with other indicators of socio-economic status [1-3]. However, there is a need for deeper understanding of the associations between parental unemployment and its consequences for children, which can be explored only if parental employment status is in the centre of the researchers' interest, not sidelined by the other socio-economic status indicators. Only a few studies solely concerning parental employment status have been published in recent years. Reinhardt Pedersen et al. [4] found increased prevalence of recurrent psychosomatic symptoms and chronic illnesses among children (aged 2–17) whose parents (one or both) were without paid work. Furthermore they found that the financial strain associated with non-employment does not explain the increased prevalence of health problems among children. Parental, particularly fathers', long-term unemployment negatively affected the subjective health of Slovak adolescents, and this negative effect remained even when adjusted for social class and financial strain [5]. Christoffersen [6] reported parental unemployment as draining children's self-esteem.

The link between parental unemployment and its consequences for adolescents could be influenced by many individual factors (e.g. intellectual ability, previous experience of unemployment, coping styles) and contextual influences (the general context in which unemployment occurs, e.g. societal settings, political situation, unemployment benefits, culture, gender role distribution). This study focuses on the contextual factors by comparing two countries with somewhat different cultural contexts: Slovakia and the Netherlands.

### Cultural context

Regarding the experience of unemployment, countries differ in many ways. There are differences in the attitudes of society to the unemployed person, the occurrence of unemployment, and the overall support for the unemployed (e.g. the duration and amount of unemployment benefits). The cultural context was suggested by Sleskova et al. [5]. They discussed the possibility that cultural environments and typical gender role distributions in different countries could be important factors influencing the association between parental unemployment and children's health. Cultural differences are visible also in terms of the effect of socio-economic status (SES) on adolescents' health. Differing results between West European countries and Central Europe have been found. For example, Tuinstra [7] found little or no SES differences in health among Dutch adolescents. On the other hand, the results of research among Slovak adolescents indicate worse health among those from lower SES [8]. Also Winefield and Fryer [9] stressed the importance of considering the historical and societal settings in which unemployment occurs.

There are several differences regarding employment status in Slovakia and the Netherlands. Firstly, the unemployment rate in these two countries is very different. In Slovakia in 1998 (time of the data collection) it was 14% while in the Netherlands in 1995 (time of the data collection) it was much lower at only 7%. There are a variety of reasons for not being employed, mainly among women. In Slovakia, the proportion of housewives is relatively small. The primary reason for being a housewife in Slovakia is usually not the need to be at home and take care of children and household, but persistent inability to find a job which results in the decision to stay at home definitely. The reasons why women do not participate in the labour market are usually maternity leave, invalidity or involuntary unemployment. The situation in the Netherlands is different. A high proportion of women consider themselves to be housewives, and being a housewife is the most frequent reason for not being employed in the Netherlands. Thirdly, unemployment benefits are much higher in the Netherlands. In Slovakia, the entitlement duration in 2003 was from 6 to 12 months. Benefits were 60% of the previous pay for the first 6 months and then 50%. In the Netherlands the duration varied from 9 months to 4 years and benefits were worth 70% of the previous pay [10]. These three differences put Slovaks without paid jobs in a less favourable position than the Dutch, and this is expected to have a greater effect on the health of Slovak children. The aim of this study was therefore to explore whether the effect of father's and mother's employment status on several aspects of adolescents' health differs between Slovakia and the Netherlands.

## Methods

### Sample

This study uses two different samples. Data from both samples were collected at schools on a voluntary and anonymous basis. Adolescents were informed about the aim of the project and signed the informed consent before filling the questionnaire. Institutional ethical committees approved the design of both studies.

The first set of data is from Slovakia and was collected in the autumn of 1998 at 31 secondary schools located in the Kosice region [8]. The sample consisted of 2616 students aged 13.8 to 17.3 years (mean age 14.9) and was stratified by type of school and gender (52% male, 48% female). Respondents completed a questionnaire at school in the absence of their teachers. A response rate of 96.3% was achieved.

The second set of data is from the Netherlands. It was collected in November 1994 and January 1995 at 18 secondary schools in all provinces of the Netherlands [11]. The sample consisted of 2054 respondents aged 14.1 to 21.8 (mean age 16.3; 50.2% male, 49.8% female). This represents a response rate of 95%. Students completed the questionnaire in their classrooms, under the guidance of a field worker.

### Measures

#### Demographics

Information about family type and respondents' education (represented by the type of school they attended) was collected.

#### Parental employment status

Parental employment status was classified differently for fathers and for mothers. For fathers, the categories employed, unemployed, disabled and retired were used. Because among women in the Netherlands being a housewife is considered to be a special position which is different from unemployment, mothers' employment status was classified into five categories: employed, unemployed, disabled, retired and housewives. As the number of retired parents was very small in our samples (for Slovak fathers 0.3% and mothers 0.2%; for Dutch fathers 1.1% and mothers 0.1%) (Table 1), this employment category was removed from all analyses.

#### Health indicators

Four health indicators representing various aspects of adolescents' subjective health were used. Because the distribution of answers was very skewed, all health indicators were dichotomised in this study.

*Self-rated health *is the one-item scale widely used in health studies as an indicator of general health status, because it is generally accepted as a good predictor of mortality and morbidity [12,13]. Respondents assessed their health using a five-point scale from "excellent" to "bad". "Excellent" and "very good" health ratings were combined into one group, and "good", "fairly good" and "bad" ratings were considered as a second group, in such a way that in this study the term "poor self-rated health" is used when referring to good, fairly good and bad ratings. This way of dichotomisation of the self-rated health scale is not common in adult samples. However, among adolescents the occurrence of "fairly good" and "bad" ratings is rare. In this study therefore we have used different cut-off point from those usually used. Only ratings 'excellent' and 'very good' have been considered as 'good self-rated health' and ratings 'good', 'fairly good' and 'bad' as 'poor self-rated health'. The same cut-off point has been used in previous studies among Slovak [5,14,15] as well as Dutch [11] adolescents.

*Psychological well-being *was measured using the 12-item version of the General Health Questionnaire (GHQ-12). The GHQ is one of the most widely-used self-report tests for assessing psychological illness [16]. The GHQ has been shown to have high validity across many different samples [17,18]. A four-point scale with binary scoring (0011) was used to answer the questions. Scores '1' were then computed as the questionnaire sum score, ranging from 0 to 12. Those having scores of 3 or more were considered as 'cases'. This cut-off point (2/3) for "cases" indicated a level of psychological distress of potential clinical significance [16,19]. In this study the term "low psychological well-being" is used when reporting the incidence of cases.

*Long-term well-being *was measured on a seven-point scale consisting of stylised faces. Respondents rated their feelings about their life in the past year. The faces were given values with number 1 meaning the best well-being and number 7 the worst. The scale was used to assess socio-emotional health in addition to global and physical health measured by other indicators. This simple scale may provide a better representation of respondents' feelings than would similar verbal scales [20]. Ratings from 1 to 3 are considered as high well-being and ratings 4 to 7 as low well-being.

*Self-esteem*. The Rosenberg Self-Esteem scale [21] is a widely-used measure of global self-esteem in adolescents. This scale consists of 10 items rated on a 4-point scale with responses ranging from "strongly agree" to "strongly disagree". The sum score ranges from 10 to 40, with 40 indicating the highest level of self-esteem. The Dutch version of the Self-Esteem measurement was a partially-modified Rosenberg questionnaire with the same number of items and sum score. For this study, the sum score was dichotomised with 25/26 as the cut-off point. To bring the scoring in line with other health indicators, it was inverted (score 26+ meaning low self-esteem).

### Statistics

The analyses were done using the statistical software package SPSS version 10.1. Logistic regression was used separately among males and females.

## Results

### Description

Table 1 presents the description of the sample. Because the school system is different in Slovakia and the Netherlands, type of school is presented separately for each country. The most frequently-attended (more than 55%) type of school in Slovakia is the four-year technical school with leaving examination (enabling students to continue study at university). The distribution of the Dutch sample is similar across all the types of school. More than 80% of both Slovak and Dutch adolescents lived with their own parents. Respondents were asked to indicate father's and mother's employment status with regard to their reasons for not being gainfully employed. The incidence of separate categories of father's employment status is very similar within the Slovak and Dutch samples. With regard to mother's employment status, a much higher proportion of housewives exists in the Dutch sample (43% compared to 5% among Slovak mothers).

Table 2 provides descriptive information about numbers and percentages of those respondents reporting ill health by gender and country. In Table 3, more detailed information about distribution of ill health can be seen. Here, attention is paid also to the gender and employment status of the parents.

Logistic regression was used to examine the relative effects of father's and mother's employment status on poor self-rated health, low psychological well-being, low long-term well-being and low levels of self-esteem among Slovak and Dutch adolescents. Firstly, only those respondents living with both parents were included in the analyses to avoid the effect of parental unemployment being confounded by the effect of family composition in one-parent families. Secondly, analyses of the whole samples were done. In the following text the results of whole-sample analyses are presented, because the odds ratios did not change considerably (not more than two decimals).

Two ways of scoring the GHQ-12 (cut-off points 1/2 and 2/3) were used in our analyses to indicate low levels of psychological well-being. Results were similar using both ways. The scoring method with the 2/3 cut-off point is used in the following text.

### Father's employment status

Table 4 presents the linkage between father's employment status and several aspects of adolescents' health.

#### Slovak

Among Slovak male adolescents, father's unemployment was a significant predictor of poor self-rated health (OR 1.81, 95% CI 1.18–2.77) and low long-term well-being (OR 2.43, 95% CI 1.36–4.34). This association was not confirmed for females. Psychological well-being and self-esteem were not associated with father's employment status, nor did having a disabled father affect any studied aspect of males' or females' health (Table 4).

#### Dutch

In the group of Dutch respondents father's unemployment was not a significant predictor of worse health status either among males or females. On the other hand, in contrast to results among Slovaks, having a disabled father increased the risk of having low psychological well-being among males (OR 2.06, 95% CI 1.05–4.01) and poor self-rated health among females (OR 2.13, 95% CI 1.01–4.46). For the other two health indicators (long-term well-being and self-esteem), no effect of father's disability on adolescents' health was found (Table 4).

### Mother's employment status

Table 5 shows the results of logistic regression with several health indicators as dependent variables and mother's employment status as independent variable.

#### Slovak

Among Slovak females, having a disabled mother increased the risk of poor self-rated health (OR 1.73, 95% CI 1.06–2.04). Among males, only self-esteem was affected by mother's employment status. The odds ratio for poor self-esteem was 1.92 (95% CI 1.01–3.61) for males whose mother was a housewife, 1.99 (95% CI 1.03–3.87) for those with disabled mothers and 1.71 (95% CI 1.03–2.84) for those with unemployed mothers compared to those with employed mothers.

#### Dutch

Among Dutch adolescents, having an unemployed or disabled mother was not a significant predictor of worse health in any of the health indicators used. On the other hand, the mother being a housewife predicted psychological well-being among females and long-term well-being among males. The mother being a housewife had a positive effect on females' (but not males') psychological well-being (OR 0.74, 95% CI 0.57–0.96). In contrast, males (but not females) whose mother was a housewife had significantly lower long-term well-being than those with employed mothers (OR 1.68, 95% CI 1.09–2.57).

## Discussion

The main aim of this study was to examine the effect of parental employment status on the health of their adolescent children in Slovakia and the Netherlands. Four main results emerged from it. Firstly, having an unemployed father negatively influences the health of Slovak male adolescents but has no effect on the health of Dutch adolescents. Secondly, having a disabled father has an effect on some aspects of the health of Dutch males and females but does not influence the health of Slovak adolescents. Thirdly, having a mother disabled, unemployed or a housewife has a negative effect on the self-esteem of Slovak adolescents. Fourthly, Dutch males whose mother was a housewife had worse long-term well being in comparison with those with an unemployed mother, whereas Dutch females whose mother was a housewife reported better psychological well-being than those with an employed mother.

One of the assumptions of the present study was that parental unemployment in Slovakia would have greater negative effects on the adolescents' health than in the Netherlands. Not all analyses supported this hypothesis, but a number of them did, mainly with regard to the fathers. One of the explanations could be the worse income circumstances in Slovakia. Men in Slovakia, whose incomes are usually higher than women's, are mostly perceived as family breadwinners. Therefore, when the father is unemployed, the family income decreases more significantly. As mentioned above, the financial support during unemployment is worse in Slovakia than in the Netherlands. Lack of financial resources has been associated with worse health in adolescence [22]. This could be the reason why father's unemployment seems to affect the health of Slovak adolescents more than Dutch ones in our research. On the other hand, our previous study on the consequences of parental unemployment [15] showed that the negative effect of father's unemployment on the health of adolescents remains even after controlling for perceived financial strain and educational level of the father. This raises the question of whether it is only finances or also a psychological effect which negatively influences adolescents' health and persists regardless of the financial situation. Stress caused by unemployment may decrease the father's support given to his children, and his frequent presence at home may increase the potential for conflicts with adolescents. Both frequent conflicts and low father's support alike have been negatively related to various aspects of health [23,24]. The present study does not allow us to answer this question. Further work should therefore be directed towards deeper understanding of the economic and psychological consequences of unemployment and their effect on the family.

Our results suggest that adolescents' health reactions to father's unemployment or disability vary not only between boys and girls within each country, but also between the studied countries. In contrast to the results from Slovakia, in the Dutch sample solely having a disabled father was negatively associated with some health outcomes. As yet, though, we have no ready explanation for this difference.

Among Dutch adolescents, two interesting associations between mother's employment status and adolescents' health were found. Having an unemployed or disabled mother had no negative effect on Dutch adolescents' health. However, females whose mothers were housewives had better psychological well-being than those with employed mothers. On the other hand, males whose mothers were housewives had worse long-term well-being than males with employed mothers. These contrasting findings need further clarification. Is it beneficial for 16-year-old adolescents to have their mother as a housewife? Do girls appreciate it, in contrast to boys? Aughinbaugh and Gittleman [25] suggest that for adolescents not having parents at home the whole day implies greater responsibility. Some of them are able to benefit from this situation and others not. Although being a housewife is very frequent in many countries, studies concerning the effect of this type of mother's daytime activity on adolescents are rare in the literature. Aughinbaugh and Gittleman [25] report no correlations between maternal employment during adolescence and increased involvement in risky activities. The daily experience of time spent with parents has been measured among early adolescents [26]. The results showed no differences in daily experience based on mothers' employment status. We are not aware of any similar study focusing on health and well-being of adolescents. Such studies would be beneficial for explaining our contrasting findings.

Despite our expectations, relationships between parental employment status and adolescents' health were not found in all health indicators and were not very strong. Previous research showed the importance of the duration of unemployment. Sleskova et al. [5] reported that parental long-term unemployment (longer than one year) has negative consequences on adolescents' health, but short-term unemployment is not important. Harland et al. [27] suggested that long-term parental unemployment has a negative effect on somatic complaints and attention problems while short-term unemployment affects behavioural and emotional problems. This indicates that the length of unemployment should also be considered in future research.

In this work we studied the effect of parental employment status on the adolescents' health, but we did not control this effect for possible confounding factors such as education, income or parental occupational status. Unemployment is often considered as an indicator of low socio-economic status [28,29]. This is due to several factors. Firstly, less-educated people and those from lower occupational classes run a greater risk of failing to find a full-time job [30]. Secondly, unemployment very often entails considerable financial loss [31] and subsequent decrease in socio-economic status. It could therefore be possible that it is not the fact that a parent is unemployed but simply low income or low parental education that affects the health of adolescents. With regard to disablement the situation may be similar. However, as mentioned before, our previous study conducted among Slovak adolescents [5] showed that low socio-economic status, represented by financial strain and low education, does not mediate the relationships between parental unemployment and health of their children. It is highly probable that such result would also be obtained in the present study, but then similar analyses would need to be made among Dutch adolescents.

Some strengths and limitations of the study should be mentioned. A key strength is that the study contains identical health and employment status indicators used in two countries and very similar samples so that it enables cross-cultural comparisons. Furthermore, the data contain closer information about the reasons for not being employed. As mentioned above, studies focusing on the trans-generational impact of parents' employment status on the health of their children are rare. Of those which have been published in recent years, we are not aware of any which has considered the reasons for not being gainfully employed. Those parents who were not employed because of retirement, disability or maternity leave, or who were housewives/househusbands, were either considered together with the unemployed as "non-employed" [4], or no information about the reasons for being without a paid job were available [5]. This work therefore adds to the knowledge about the effects of parental employment status by distinguishing between the reasons for parents not being gainfully employed.

The first limitation of this study is the categorization of mothers' employment status. The category 'housewife' is rather problematic for Slovak adolescents. As mentioned above, women become housewives in Slovakia mainly because of their long-term inability to find a job. So it may happen that adolescents whose mother is actually housewife and is not looking for the job anymore indicated her to be unemployed or vice versa. However, according to our results, it seems to be that with regard to health of children, being unemployed or being a housewife has the same effect. Therefore we believe it is unlikely that this limitation will bias our study.

The second limitation is the difference between the mean ages of the studied cohorts, which is somewhat more than one year. Even though we tried to make our data sets as comparable as possible, the age of the respondents is slightly different. Given the fact that both studied samples were selected from secondary school students, living in most cases with their parents, the age difference should not be so problematic. Furthermore, the age difference can only explain our results if the SES gradient depends on the adolescents' age, and this age effect would have to be very large to explain our results.

## Conclusion

Despite these limitations the present study contributes to the knowledge about the relationships between parental employment status and adolescents' subjective health. It enables us to compare the effect of father's and mother's employment status on their children's health in Slovakia and the Netherlands, and it pays closer attention to the reasons for parents not being gainfully employed. To conclude briefly our results, father's unemployment seems to be a better predictor of health for Slovak adolescents, father's disablement of health for Dutch ones. Mother's employment status seemed to be important for the self-esteem of Slovak adolescents and mother as a housewife for the health of Dutch ones. Our study shows that the effect of parental employment status could be different in various countries, and indicates therefore that further studies from other cultures would be useful.

## Competing interests

The author(s) declare that they have no competing interests.

## Authors' contributions

Every author participated in the writing and reviewing the drafts of the manuscript. AMG, JPvD, JT and JWG are responsible for the design of the studies; MS and JT contributed to the conception and design of the paper; MS, SAR, FS and JT contributed to the acquisition and analysis of the data, and to the interpretation of the results; MS, JT, AMG, JPvD, JWG and SAR drafted and revised critically the paper. All authors read and approved the final version of manuscript.

## Pre-publication history

The pre-publication history for this paper can be accessed here:



## Figures and Tables

**Table 1:** Descriptive characteristics of the sample.

		Slovak % (N)	Dutch % (N)
Gender	male	52.4 (1370)	50.2 (1032)
	female	47.6 (1246)	49.8 (1022)
			
Mean age (SD)		14.9 (0.62)	16.3 (0.75)
			
Type of school ^a^	grammar school (4 years)	21.8 (571)	
	technical school (4 years)	55.4 (1450)	
	apprentice school (2 or 3 years)	22.8 (595)	
			
Type of school	pre-university education (6 years)		21.3 (437)
	higher secondary education (5 years)		26.1 (537)
	lower secondary education (4 years)		25.5 (523)
	vocational education (4 years)		22.6 (465)
	individual education		4.5 (92)
			
Family type	both own parents	85.0 (2224)	83.5 (1715)
	only one own parent	8.1 (211)	9.6 (197)
	one own parent + one step parent	4.5 (119)	3.5 (72)
	other	0.1 (2)	3.4 (70)
	missing	2.3 (60)	0
			
Father's ES	employed	85.1 (2226)	86.5 (1777)
	unemployed	7.4 (193)	3.8 (78)
	disabled	3.3 (87)	3.7 (77)
	retired	0.3 (9)	1.1 (22)
	househusband/maternity leave	0.0 (1)	1.0 (20)
	missing	3.8 (100)	3.9 (80)
			
Mother's ES	employed	79.5 (2079)	49.5 (1016)
	unemployed	8.7 (228)	3.6 (74)
	disabled	5.1 (134)	2.6 (53)
	retired	0.2 (5)	0.1 (3)
	housewife/maternity leave	5.4 (141)	42.6 (875)
	missing	1.1 (29)	1.6 (33)

^a^Because of the different school systems in Slovakia and the Netherlands, type of school is presented separately for each country.

ES = employment status.

**Table 2:** Prevalence of adolescents with worse health by gender and country of origin.

	Slovak		Dutch	
	
	Males % (N)	Females % (N)	Males % (N)	Females % (N)
Poor self-rated health	31.1 (424)	44.9 (558)	37.2 (384)	56.4 (574)
Low psychological well-being	24.3 (321)	43.6 (433)	22.1 (221)	43.6 (433)
Low long-term well-being	8.8 (119)	10.4 (129)	10.3 (105)	14.8 (150)
Low self-esteem	13.0 (171)	24.8 (302)	27.2 (280)	44.1 (448)

**Table 3:** Prevalence of adolescents with worse health by gender, country of origin of resondents and gender and employment status of parents.

**Father's ES**		Slovak		Dutch	
		Males (%)	Females (%)	Males (%)	Females (%)

**Poor self-rated health**	Employed	30.0	43.7	37.2	55.0
	Unemployed	43.6	51.5	32.5	67.6
	Disabled	27.9	56.8	36.6	72.2
					
**Low psychological well-being**	Employed	24.2	42.1	20.8	43.0
	Unemployed	28.4	42.9	24.3	56.8
	Disabled	24.4	39.5	35.0	42.4
					
**Low long-term well-being**	Employed	7.9	10.5	9.9	13.8
	Unemployed	17.2	9.3	10.0	23.7
	Disabled	11.6	6.8	14.6	19.4
					
**Low self-esteem **	Employed	12.5	23.9	27.2	44.4
	Unemployed	18.7	26.3	22.5	48.6
	Disabled	14.3	31.8	26.8	41.7

**Mother's ES**					

**Self-rated health**	Employed	31.0	44.3	36.2	58.3
	Housewife	31.5	44.1	38.7	54.0
	Unemployed	28.0	42.2	30.3	57.5
	Disabled	35.4	58.0	44.0	53.6
					
**Psychological well-being**	Employed	24.4	42.6	23.0	47.0
	Housewife	21.7	34.3	20.2	39.5
	Unemployed	22.2	38.5	37.5	47.4
	Disabled	26.3	38.8	20.0	53.6
					
**Long-term well-being**	Employed	8.8	10.2	8.0	12.4
	Housewife	10.1	2.9	12.7	16.7
	Unemployed	7.6	12.1	15.2	22.0
	Disabled	10.8	16.4	12.0	15.4
					
**Self-esteem **	Employed	11.6	24.2	25.7	45.0
	Housewife	20.0	21.2	29.7	44.3
	Unemployed	18.3	30.5	24.2	43.9
	Disabled	20.7	27.3	20.0	35.7

**Table 4:** The effect of fathers' employment status on adolescents' health (logistic regression; odds ratios, 95% confidence intervals)

**Father's ES**	Slovak		Dutch	
	
	males OR (95% CI)	females OR (95% CI)	males OR (95% CI)	females OR (95% CI)
**Self-rated health**				
Employed	1.00	1.00	1.00	1.00
Unemployed	**1.81 **(1.18–2.77)	1.37 (0.91–2.08)	0.81 (0.41–1.60)	1.71 (0.85–3.44)
Disabled	0.91 (0.46–1.78)	1.70 (0.92–3.12)	0.97 (0.51–1.87)	**2.13 **(1.01–4.46)
**Psychological well-being**				
Employed	1.00	1.00	1.00	1.00
Unemployed	1.24 (0.75–2.05)	1.03 (0.68–1.57)	1.23 (0.57–2.65)	1.74 (0.90–3.38)
Disabled	1.01 (0.49–2.08)	0.90 (0.48–1.68)	**2.06 **(1.05–4.01)	0.98 (0.48–1.97)
**Long-term well-being**				
Employed	1.00	1.00	1.00	1.00
Unemployed	**2.43 **(1.36–4.34)	0.87 (0.43–1.78)	1.01 (0.35–2.90)	1.94 (0.90–4.21)
Disabled	1.54 (0.59–4.01)	0.62 (0.19–2.05)	1.55 (0.64–3.80)	1.51 (0.65–3.53)
**Self-esteem**				
Employed	1.00	1.00	1.00	1.00
Unemployed	1.62 (0.93–2.82)	1.14 (0.71–1.84)	0.78 (0.36–1.65)	1.19 (0.61–2.29)
Disabled	1.71 (0.48–2.83)	1.49 (0.78–2.85)	0.98 (0.48–1.99)	0.89 (0.45–1.76)

Significant effect is in bold

**Table 5:** The effect of mothers' employment status on adolescents' health (logistic regression; odds ratios, 95% confidence intervals)

**Mother's ES**	Slovak		Dutch	
	
	males OR (95% CI)	females OR (95% CI)	males OR (95% CI)	females OR (95% CI)
**Self-rated health**				
Employed	1.00	1.00	1.00	1.00
Housewives	1.02 (0.62–1.71)	0.99 (0.61–1.63)	1.11 (0.86–1.45)	0.84 (0.65–1.09)
Unemployed	0.86 (0.57–1.32)	0.92 (0.62–1.37)	0.77 (0.36–1.65)	0.97 (0.51–1.86)
Disabled	1.22 (0.72–2.06)	**1.73 **(1.06–2.84)	1.39 (0.62–3.12)	0.83 (0.39–1.77)
**Psychological well-being**				
Employed	1.00	1.00	1.00	1.00
Housewives	0.86 (0.46–1.61)	0.71 (0.42–1.19)	0.85 (0.62–1.16)	**0.74 **(0.57–0.96)
Unemployed	0.89 (0.55–1.43)	0.85 (0.56–1.27)	2.01 (0.95–4.24)	1.02 (0.53–1.97)
Disabled	1.11 (0.61–2.03)	0.86 (0.52–1.42)	0.84 (0.31–2.28)	1.30 (0.61–2.80)
**Long-term well-being**				
Employed	1.00	1.00	1.00	1.00
Housewives	1.17 (0.52–2.62)	0.27 (0.07–1.12)	**1.68 **(1.09–2.57)	1.41 (0.98–2.03)
Unemployed	0.85 (0.42–1.74)	1.22 (0.66–2.26)	2.06 (0.76–5.63)	1.98 (0.90–4.35)
Disabled	1.25 (0.55–2.81)	1.74 (0.88–3.42)	1.58 (0.45–5.49)	1.28 (0.43–3.84)
**Self-esteem**				
Employed	1.00	1.00	1.00	1.00
Housewives	**1.91 **(1.01–3.61)	0.84 (0.46–1.55)	1.22 (0.92–1.62)	0.97 (0.75–1.26)
Unemployed	**1.71 **(1.03–2.84)	1.37 (0.88–2.13)	0.92 (0.41–2.10)	0.96 (0.51–1.82)
Disabled	**1.99 **(1.03–3.87)	1.17 (0.67–2.06)	0.72 (0.27–1.96)	0.68 (0.31–1.50)

Significant effect is in bold
